# A systematic review of ecological attributes that confer resilience to climate change in environmental restoration

**DOI:** 10.1371/journal.pone.0173812

**Published:** 2017-03-16

**Authors:** Britta L. Timpane-Padgham, Tim Beechie, Terrie Klinger

**Affiliations:** 1 School for Marine and Environmental Affairs, University of Washington, Seattle, Washington, United States of America; 2 Ocean Associates Inc., under contract to Northwest Fisheries Science Center, National Marine Fisheries Services, National Oceanic and Atmospheric Association, Seattle, Washington, United States of America; 3 Fish Ecology Division, Northwest Fisheries Science Center, National Marine Fisheries Service, National Oceanic and Atmospheric Association, Seattle, Washington, United States of America; University of Sydney, AUSTRALIA

## Abstract

Ecological restoration is widely practiced as a means of rehabilitating ecosystems and habitats that have been degraded or impaired through human use or other causes. Restoration practices now are confronted by climate change, which has the potential to influence long-term restoration outcomes. Concepts and attributes from the resilience literature can help improve restoration and monitoring efforts under changing climate conditions. We systematically examined the published literature on ecological resilience to identify biological, chemical, and physical attributes that confer resilience to climate change. We identified 45 attributes explicitly related to climate change and classified them as individual- (9), population- (6), community- (7), ecosystem- (7), or process-level attributes (16). Individual studies defined resilience as resistance to change or recovery from disturbance, and only a few studies explicitly included both concepts in their definition of resilience. We found that individual and population attributes generally are suited to species- or habitat-specific restoration actions and applicable at the population scale. Community attributes are better suited to habitat-specific restoration at the site scale, or system-wide restoration at the ecosystem scale. Ecosystem and process attributes vary considerably in their type and applicability. We summarize these relationships in a decision support table and provide three example applications to illustrate how these classifications can be used to prioritize climate change resilience attributes for specific restoration actions. We suggest that (1) including resilience as an explicit planning objective could increase the success of restoration projects, (2) considering the ecological context and focal scale of a restoration action is essential in choosing appropriate resilience attributes, and (3) certain ecological attributes, such as diversity and connectivity, are more commonly considered to confer resilience because they apply to a wide variety of species and ecosystems. We propose that identifying sources of ecological resilience is a critical step in restoring ecosystems in a changing climate.

## Introduction

Substantial degradation of earth’s ecosystems—and powerful legal mandates such as the U.S. Endangered Species Act, U.S. Clean Water Act, E.U. Water Framework Directive, and E.U. Habitats Directive—have led many governmental agencies, non-profit organizations, and private interest groups to invest in restoration efforts. This ‘restoration economy’ was recently estimated to contribute $24.86 billion and 221,000 jobs annually to the U.S. economy [1]. Yet despite such monumental investments, ecological restoration has often been unsuccessful in reducing extinction rates and slowing declines in habitat quality [2–5]. On the other hand, evidence of increased biodiversity and improved ecosystem function following restoration demonstrates that restoration can be successful in rehabilitating the condition of ecosystems [5, 6], and restoration now serves as an accepted and widely practiced management action.

Ecological restoration proceeds in the face of advancing climate change, which imposes additional stress on systems already under pressure from human use and this can undermine the long-term success of restoration efforts [7]. To address this concern, many have suggested a shift away from static restoration end points and towards dynamic and adaptive ecological process goals [3, 8, 9]. Evidence suggests that climate change impacts on populations and communities are increasingly considered in the development of management priorities and adaptation plans. For example, recent climate change studies have utilized trait-based vulnerability assessments to identify both potential impacts and inherent natural sources of climate-change resilience for individual species [10–14]. These assessments have in turn informed the development of decision-support frameworks to incorporate climate change into restoration planning [15, 16].

Integrating resilience concepts and attributes could help improve restoration and monitoring efforts under conditions of climate change. Resilience approaches to restoration can foster adaptation to future climate impacts [15, 17–19] by restoring dynamic processes that promote natural variability and biodiversity within ecological systems, and reducing the risk of dramatic ecosystem change, sharp declines in populations, or loss of ecosystem services [20–22]. Ecological resilience incorporates concepts of dynamic feedbacks, unpredictable change, and variation [23, 24]. Here we use the resilience perspective of Walker et al. [25] that defines resilience as the capacity of a system to absorb disturbance and reorganize in ways that retain essentially the same functions, structures, identities, and feedbacks. This definition includes two important mechanisms of resilience, namely resistance to change and recovery from change.

To understand how resilience attributes can be integrated into restoration practices under climate change, we first distilled common attributes of ecological resilience from the published literature. We then applied a ‘climate filter’ to identify attributes likely to confer resilience under changing climate conditions. We further classified these attributes according to their ecological scale of application. We provide three examples to illustrate how practitioners can select resilience attributes that are appropriate for specific management applications. Finally, we outline general strategies for integrating resilience into restoration planning and monitoring in a changing climate.

## Methods

### Literature selection and examination

We examined the scientific literature to extract attributes of species or ecosystems that have been reported to confer ecological resilience. Using Web of Knowledge, one of us (BLTP) searched using the following terms: (river* OR stream OR (wetland NOT in title) OR ecosystem OR environment*) AND (restor* OR recov* OR re-creat* OR rehabilitat*) AND (resilienc* OR “ecological integrity”), restricting our search to papers published from 2009–2013. From a total of 915 search results, 232 articles were selected for further examination if the title described a scientific study investigating the resilience of some ecological characteristic(s). Of the 232 articles, 111 were selected for full review based on relevance to the study objectives as inferred from the abstract. Fifty-nine additional articles were gleaned from the selected literature based on best professional judgment of their fit with the goals of this study. These articles were added to the analysis for a total of 170 articles examined in this study (S1 Fig). For consistency, and to reduce inter-observer variation, all examination of the literature was performed by BLTP.

### Resilience attribute identification

Attributes of ecological resilience were selected for further consideration if they were (1) typical of more than one ecosystem or species, (2) distinct from other attributes, and (3) measureable. From the assembled attributes, we created a database in which every attribute from each publication was recorded, along with the source of publication, ecosystem context, metric(s) used to measure or monitor the attribute, and whether the attribute was identified as conferring resistance to or recovery from disturbance. We then grouped the attributes into major categories and combined attributes that were similar to produce a list of 51 resilience attributes classified into five major categories. The resilience attributes that we identified come from a wide-range of ecosystems and range from more general (e.g. energy flows) to more specific (e.g. soil and air carbon balance). Given that our primary purpose in this study was to broadly inform restoration practices under climate change, we elected to retain as many attributes as possible and to broadly define terms to maximize utility to practitioners working across a range of scales and contexts. Practitioners can choose to further refine attributes and definitions based on specific applications.

### Climate change filter

We next evaluated the attributes to identify those that were considered to confer resilience to climate change. An attribute passed through the climate change filter if the article specifically mentioned an attribute in relation to climate change or climate impacts. For example, if the article discussed how an attribute might confer resilience to climate change or an ecological feature directly affected by climate change such as stream flow or temperature, the attribute was retained in our list of resilience attributes. A total of 45 (out of 51) attributes remained after the climate filter was applied. Attributes eliminated by the climate filter (population (beta) diversity, gamma diversity, food-web complexity, large woody debris, salinity, and historical flow-disturbance regimes) may confer resilience to climate change impacts in some situations, but that was not apparent in the articles evaluated.

### Attribute classification

We classified the 45 resilience attributes from our review into five categories that roughly equate to ecological scale: (1) individual attributes, (2) population attributes, (3) community attributes, (4) ecosystem attributes, and (5) process attributes. We used best professional judgment to classify each attribute by two criteria that we felt were integral for any restoration project: restoration focus (e.g., is the restoration effort species-specific, habitat-specific, or system-wide focused?) and scale of application (e.g., do restoration actions take place at a population, site, or ecosystem scale?). ‘Restoration focus’ refers to the *type* of project an attribute is best suited for. For example, a population attribute such as density is likely more suitable for a restoration effort that aims to restore a species, whereas a community attribute such as functional diversity is more applicable to a restoration effort aiming to restore an ecosystem. Some attributes were assigned to more than one category because they are suitable for more than one restoration focus. ‘Scale of Application’ denotes the scale an attribute can be used to describe (e.g., generally population scales for biological attributes, and site or ecosystem scales for environmental attributes). Several attributes were assigned to more than one scale because scale varies depending on environmental context or project type. Our classification does not account for every potential application; consequently, users may need to tune some classifications to meet the needs of particular systems or projects.

In a practical sense, the resilience attributes all serve as ecological metrics that can be used for monitoring efforts (e.g. population size, presence of propagules, recovery time after disturbance) and/or setting ecological goals for restoration projects (e.g. genetic diversity, increase or establish refugia or support areas, release from competition or predation).

### Attribute selection and sample applications

The attribute classifications can be used to create a decision support table (DST) by using a filtering function (S1 Table) so that practitioners can identify resilience attributes that are best suited to the focus and spatial scale of a specific restoration plan or project. To create a sub-list of suitable resilience attributes, a practitioner can sort attributes by asking: (1) what is the focus of the restoration project? and (2) what is the spatial scale of the specific needs? The output comprises a sub-set of resilience attributes that are more likely to be relevant to the specific plan or project.

To illustrate use of the DST in restoration planning, we created three sample applications. We selected three different restoration efforts focused at different spatial scales to demonstrate (1) how relevant resilience attributes can be identified for a specific project and (2) how the attributes selected will differ according to the type of project. We use the Kissimmee watershed system as an example of restoration at the ecosystem scale, a Pacific salmon (*Oncorhynchus* spp.) population as an example of restoration at the population scale, and vulnerable coral species as an example of restoration at the site scale.

## Results and discussion

### Summary of literature examined

**Fig 1:**
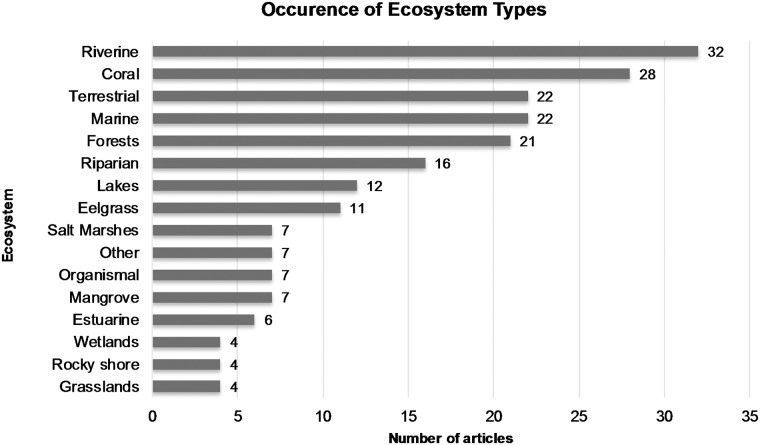
Frequency distribution of articles by ecosystem type.

Most articles referred to riverine and coral ecosystems (32 and 28 citations, respectively), followed by terrestrial, marine, and forest ecosystems (Fig 1). Rocky shore, wetland, and grassland ecosystems were less commonly cited (4 citations each). While our search terms did include river or stream habitats, as that was our intended focus originally, we also include broader terms of ‘ecosystem’ or ‘environment’ which resulted in a diverse representation of habitat types. The number of times an individual attribute was cited varied from 1–20. By attribute type, ecosystem attributes were most frequently cited (Table 1), but there were more total citations of process attributes (63) because more than one third of all attributes (16, or 36%) were classified as process attributes.

**Table 1:** Resilience attribute table.

	Resilience Attribute Category	Grouped Attributes	Resistant	Recovers	Times Cited	Sources
**Biological Attributes**	**Individual Attributes**	**Individual Growth rate**		X	4	[26–29]
		**Individual Size**	X	X	7	[27, 30–35]
		**Life span**	X	X	4	[28, 36–38]
		**Individual characteristics that favor flexibility or adaptability**		X	10	[37, 39–47]
		**Reproductive Strategy**	X	X	4	[26, 42, 46]
		**(Biological) adaptation to disturbance**	X	X	14	[27, 33, 36, 40, 41, 43–46, 48–52]
		**Presence of propagules**		X	5	[52–56]
		**Dispersal Potential**	X	X	10	[18, 28, 33, 36, 38, 42, 47, 57–59]
		**Efficient water capture and use**	X		2	[60, 61]
	**Population Attributes**	**Genetic Diversity**	X	X	9	[22, 36, 62–68]
		**Population Size**	X	X	8	[22, 36, 46, 67–71]
		**Population Density**	X	X	10	[23, 30, 43, 69, 70, 72–76]
		**Population Growth Rate**		X	1	[38]
		**Population Age structure**	X	X	4	[66, 69, 71, 77]
		**Connectivity Between Populations**		X	8	[36, 67, 72, 78–82]
		**Population (Beta) Diversity**	X		2	[83, 84]
	**Community Attributes**	**Community Structure**	X	X	4	[35, 61, 72, 85]
		**Species Assemblage**		X	15	[3, 8, 24, 27, 31, 36, 39, 58, 86–92]
		**Species (Alpha) Diversity**	X	X	17	[5, 7, 20, 36, 39, 60, 61, 86, 93–101]
		**Functional Diversity**	X	X	10	[19, 61, 102–109]
		**Response Diversity**	X	X	7	[36, 103, 106, 110–113]
		**Functional Redundancy**	X	X	8	[36, 103, 105, 113–117]
		**Connectivity among communities**	X	X	7	[36, 75, 82, 118–121]
		**Gamma Diversity**	X	X	2	[19, 122]
**Physical Attributes**	**Ecosystem Attributes**	**Habitat Area**	X	X	6	[19, 72, 79, 123–125]
		**Habitat Structure**	X	X	7	[36, 72, 85, 106, 110, 126, 127]
		**Habitat Condition**	X	X	14	[31, 32, 60, 75, 86, 124, 128–135]
		**Temporal Variability in Habitats**	X	X	10	[20, 48, 87, 120, 124, 136–140]
		**Spatial Variability in Habitats**	X	X	20	[17, 20, 27, 33, 48, 49, 60, 74, 79, 82, 86, 87, 120, 123, 124, 129, 137–139, 141]
		**Refugia or Support Areas**	X	X	13	[20, 29, 33, 34, 39, 43, 66, 74, 86, 87, 124, 142, 143]
		**Connectivity between different Habitats**	X	X	18	[19, 36, 39, 66, 75, 79, 80, 82, 86, 119, 120, 123, 144–149]
		**Food Web Complexity**	X		2	[126, 150]
		**Large Woody Debris (LWD)**	X	X	1	[151]
		**Salinity**		X	1	[152]
	**Process Attributes**	**Connectivity to refugia areas**		X	5	[33, 39, 52, 91, 153]
		**Energy Flows**	X	X	12	[30, 39, 40, 87, 126, 128, 152, 154–158]
		**Natural release from competition or predation**	X	X	3	[36, 157, 159]
		**Sedimentation**	X	X	4	[33, 129, 160, 161]
		**Soil and Air Carbon Balance**		X	2	[72, 130]
		**Hyporheic Flows**	X		2	[151, 162]
		**Flow Regime**	X	X	4	[18, 37, 87, 163]
		**Groundwater Contributions**	X		2	[74, 124]
		**Structural legacies**		X	9	[31, 36, 47, 54–56, 143, 164, 165]
		**Water Infiltration**	X	X	3	[2, 104, 136]
		**Feedback between physical and biological processes**	X	X	2	[129, 166]
		**Recovery (time) after disturbance**		X	5	[43, 52, 91, 127, 167]
		**Natural disturbance history**	X	X	15	[24, 39, 44, 52, 57, 69, 73, 130, 138, 154, 156, 164, 168–170]
		**Random environmental variability**	X	X	6	[26, 42, 82, 120, 137, 171]
		**Disturbance duration and intensity**		X	12	[18, 24, 31, 39, 52, 56, 88, 104, 127, 152, 172, 173]
		**Degree of exposure to human pressures**		X	5	[8, 55, 69, 139, 174]
		**Historical flow-disturbance regimes**	X		4	[58, 89, 175, 176]

Each resilience attribute is listed and grouped into five major categories (Individual, Population, Community, Ecosystem, and Process) and whether the attribute was identified as resisting or recovering from disturbance in the literature is noted. In addition, the number of citations for each attribute and the corresponding references are detailed. Attributes highlighted in grey did not pass through the climate filter.

More than half (33 of 45) of the resilience attributes were defined as equivalent to resistance (to perturbation), and many others (42 of 45) used resilience synonymously with recovery, or as an outcome of recovery (Table 1). Across all studies, 30 of the 45 attributes were used in both ways (i.e., some studies considered resilience to mean recovery, while others considered it to mean resistance). However, only a few sources explicitly considered resilience to include both concepts: that of resistance, or the ability of an ecosystem or community to persist through a disturbance, and that of recovery, or rate at which a system or community returns to its functional state.

Several studies in our review consisted of a census of resilience attributes within a specific ecosystem type [15, 51, 54, 58, 66, 113, 126]. Maynard et al. [66] used a literature review to distill a list of 19 ‘resilience indicators’ that ‘conferred resilience’ within coral reef systems. In a study by McClanahan et al. [113], a group of 50 scientists ranked and scored an existing list of ‘resilience factors’ also in coral reef systems. Bernhardt and Leslie [126] conducted a comprehensive study exploring sources of resilience to climate change within coastal marine ecosystems and found three important ecological themes for conferring resilience: connectivity, biological diversity, and adaptability. Our review, which included the aforementioned studies, also found these three ecological themes to be widely cited in the literature, but to these we add habitat variability and condition, presence of refugia or support areas, and natural disturbance history as commonly-cited themes. We discuss these themes and the influence of human pressures on resilience in the following sections.

### Connectivity

Connectivity was found to enhance capacity for self-organization and recovery at multiple scales, including interactions between species at community and population scales and connectivity of habitat types and ecosystems throughout both space and time [126]. Connectivity supports resilience by allowing movement of propagules, larvae and adults to recolonize a disturbed area or replenish an area with new genetic material and enhance local populations. Fritz and Dodds [42] observed how flooding events increasing invertebrate populations by connect intermittent pools in rivers and provided for colonization and dispersed young individuals. In coral reef systems, Olds et al. [78] found that connectivity between mangrove ecosystems and protected reefs in eastern Australia enhanced herbivore biomass and richness.

Connectivity of various healthy habitat types helps maintain species that use a variety of habitats for feeding, reproduction, resting, rearing, refuge, and migrating [51]. In riverine systems, ecological connectivity is important for maintaining natural variability and supporting productivity [102]. Many species, such as Pacific salmon, rely on movement throughout the system, including the mainstem, tributaries, floodplain habitats, and deltas. Removal of anthropogenic barriers to migration can help increase resilience of aquatic biota to climate change impacts such as changing flow regimes [151]. Ecosystem connectivity is also critical to help regulate essential abiotic and biotic processes such as flow, temperature, water quality, aquatic and terrestrial interactions and food webs.

### Biodiversity and the insurance hypothesis

Alpha diversity, genetic diversity, and functional diversity were the most frequently cited diversity attributes. Duffy [154] found that on average, greater species richness increased resource use within trophic levels and accumulation of biomass, and that the variance in these responses was reduced over time. Moreover, diverse communities have a higher chance of including either disturbance-resistant species or species that are able to recover quickly from a variety of perturbations [126, 176]. Ecosystems or communities with greater functional and response diversity are able to maintain important ecosystem processes that sustain function and result in ‘no net loss’ in productivity, often referred to as the insurance hypothesis [94, 116, 147, 164]. In an experimental study Naeem and Li [147] tested the hypothesis that a greater number of species should enhance the probability that a system will provide a more “consistent level of performance” using microbes. They found that the greater number of species per functional group led to more consistent biomass and density measures within the replicated microbial microcosms. Genetic diversity can provide this benefit by increasing the critical response diversity among populations and can help maintain ecosystem function [112, 126]. Additionally, increased genetic diversity has been shown to promote population growth and improve fitness [177].

There is ongoing debate over the association between biodiversity and its influence on resilience. Not all findings support the insurance hypothesis. For example, in a greenhouse experiment, Lanta et al. [57] found that high species richness and functional diversity provided less resistance against drought stressed conditions than less diverse species assemblages. The same study found no effect of diversity on community resistance under outdoor experimental conditions. Similarly, in a study examining species richness in aquatic food webs, Downing and Leibold [63] found that while respiration rates showed “higher resilience” in species-rich communities, they did not exhibit increased “resistance” to disturbance. In contrast, however, a number of studies have found strong causal linkages between diversity measurements and productivity or stability in a number of terrestrial and aquatic systems [154], including seagrass [130, 136] and forests [38, 178].

### Habitat variability and condition

Spatial and temporal variability in habitats have been observed to maintain higher levels of biodiversity [94], and thus contribute to ecosystem resilience. A study conducted by Oliver and others [64] found landscape structure, including increased heterogeneity within habitat patches, to influence resilience of populations to extreme climatic events. A landscape with a more heterogeneous habitat structure was more likely to contain refuge microclimates to support survival of the ringlet butterfly, and greater heterogeneity among habitat patches increased the likelihood of harboring species more resilient to extreme events [64]. Within river systems, spatiotemporal variability in flow and temperature regimes was found to regulate suitable habitat and maintain flexible species adaptations [58, 67, 79]. Milner et al. [151] showed that maintaining habitat heterogeneity can maximize resilience of aquatic species to altered flow regimes associated with climate change. While habitat variability generally increases diversity at various scales, it also serves as a useful “measure of resilience to impending climate change” [165].

### Refugia and support areas

Within the ecosystem category, presence of refugia or support areas was particularly important to ecosystem resilience. In freshwater and salt marsh ecosystems, presence and type of riparian vegetation was found to create micro-habitats that promoted community resistance to dry conditions [35, 62, 80]. Various soil health metrics were identified as crucial for aiding in recovery of forest ecosystems [107, 132] and improving functional resilience in other terrestrial ecosystems [179–181]. Studies in coral reef systems identified water quality to be an important control on macroalgal growth, which can cause serious negative impacts to coral recruitment and overall reef resilience [59, 78, 135]. Refugia can also serve as areas where species are able to survive or rest from disturbance [19, 29, 55, 67, 68, 79, 82, 141], and have been documented to provide propagules or seed sources for recovery in other affected areas [26, 28, 37, 151, 182]. These particular habitat attributes may not influence resilience in every ecosystem, but these findings suggest that identifying principal habitat characteristics may be an important consideration in monitoring resilience within an ecosystem.

### Natural disturbance history and adaptability

A history of natural environmental fluctuations and disturbance is one process that maintains habitat heterogeneity, and the variability induced by disturbances favors biodiversity [94]. Specifically, disturbance can regulate habitat structure at multiple scales, with the potential to affect species richness many years into the future [52, 83, 86, 107]. A substantial proportion of the literature identified presence of natural disturbance as an important determinant for recovery rates, creation of alternate trajectories, and building biological capacity to adapt to or resist change. Systems that are naturally subjected to a variety of disturbances contain biota that have evolved life history traits favoring adaptability or flexibility [61, 114, 182]. Li et al. [61] determined that bacterioplankton communities in a lake ecosystem had developed a number of life history attributes (e.g., high growth rates, phenotypic flexibility) that favored adaptation and explained their high resilience to natural pulses of Microcystis blooms. Within marine ecosystems, Neubauer et al. [45] confirmed that a history of moderate exploitation within fisheries populations can increase their rate of recovery.

Natural disturbance can influence biophysical characteristics of ecosystems and populations. For example, the size of a disturbed area can influence recovery rates because it effects how close it is to undisturbed neighboring areas that can provide material for re-colonization [162]. Some authors characterized entire ecosystems that are subject to high levels of natural disturbance as resilient. The hypothesis is that systems with high levels of disturbance have adapted with species and or processes that support quick recovery or resist complete change altogether [7, 19, 75, 83, 90, 182, 183]. In addition to disturbance, the magnitude and duration of an event proved to be an important attribute conferring resilience within many different systems. A number of studies found disturbance intensity to affect the degree of recovery [136, 149] with more severe disturbance being a predictor of more rapid recovery [48, 169]. Despite many systems demonstrating a considerable resilience to disturbance, prolonged disturbance is more likely to result in persistent habitat changes and reduce the ability of a system or populations to recover [175]. There is also considerable concern about future impacts on disturbance duration, magnitude, frequency, and timing from human induced climate change [149, 166].

The effects of increased disturbance due to climate change do pose serious unknowns for resilience. For example, holm oak woodlands are historically highly resilient to fire frequencies of about 50 year intervals, but if the frequency of fire increases in response to climate change the system may not exhibit the same degree of resilience [149]. In a study examining resilience of fishes and invertebrates in streams exposed to prolonged drought, Bêche and others [175] found that both severity and duration of drought disturbance influenced the abundance, richness, and general recovery of aquatic communities.

### Human pressures, cumulative effects

We found contradictory evidence regarding the effects of human pressures on resilience. A number of studies reported that isolation from human pressures or reduced exposure to anthropogenic stressors increased resilience within their systems [33, 121, 133]. Alternatively, in a study of coral assemblages distributed over a wide geographic range, Côté and Darling [54] found that *if* there is a positive co-tolerance between non-climatic disturbance and climatic impacts among coral species, then some degree of human-caused degradation may “increase the abundance of disturbance-tolerant species within a community and thus the ability of an ecosystem to resist impacts of climatic disturbance”. However, reduced abundance of less tolerant species (and increased proportions of disturbance-tolerant species) can also be considered an indicator of ecosystem degradation, at least in some contexts [184].

A number of resilience attributes we identified, including exposure to human pressures, were often discussed in context of cumulative impacts. This is an important consideration when measuring resilience in locations subject to multiple human stressors. The ability of ecosystems and their components to maintain resilience in the face of climate change when those systems are already under stress from cumulative human-generated impacts is a topic of evident concern in the literature [3, 54, 159]. Multiple co-occurring modes of disturbance can confound efforts to identify, measure, and monitor resilience within a system.

### Restoration examples using the DST

Attributes classified by restoration focus and scale of measurement roughly sorted according to attribute category (Table 2). For example, individual and population attributes (e.g., dispersal potential or genetic diversity) tended to be associated with species-specific restoration actions and with resilience at the population scale. Community attributes generally described the structure and diversity of ecosystems (e.g., community structure, functional diversity, or species diversity), and therefore were most often associated with site-specific or system-wide restoration. Roughly half of the ecosystem attributes (e.g., habitat area and condition, or refuge areas) were associated with all three restoration foci and at all three spatial scales. Process attributes were most diverse with respect to both focus and scale.

**Table 2:** Decision Support Table (DST).

	Resilience Attributes	Restoration Focus (species, habitat, system)	Scale of Application (population, site, ecosystem)
**Individual Attributes**	Individual growth rate	species	population
	Individual size	species	population
	Life span	species	population
	Individual characteristics that favor flexibility or adaptability *	species, habitat	population, site
	Reproductive strategy	species	population, site
	(Biological) Adaptation to disturbance *	species, habitat	population, site, ecosystem
	Presence of propagules	species, habitat	population, site
	Dispersal potential *	species, habitat	population, site
	Efficient water capture and use	species, habitat	population, site
**Population Attributes**	Genetic diversity *	species, habitat	population
	Population size *	species	population
	Population density *	species, habitat	population
	Population growth rate	species	population
	Population age structure	species	population
	Connectivity between populations*	species	population, ecosystem
**Community Attributes**	Community structure	habitat, system	site, ecosystem
	Species assemblage *	habitat, system	site, ecosystem
	Species (alpha) diversity *	habitat, system	site, ecosystem
	Functional diversity *	habitat, system	site, ecosystem
	Response diversity	habitat, system	site, ecosystem
	Functional redundancy *	habitat, system	site, ecosystem
	Connectivity among communities	habitat, system	ecosystem
**Ecosystem Attributes**	Habitat area	species, habitat, system	population, site, ecosystem
	Habitat structure	species, habitat, system	site, ecosystem
	Habitat condition *	species, habitat, system	population, site, ecosystem
	Temporal variability in habitats *	system	ecosystem
	Spatial variability in habitats *	habitat, system	ecosystem
	Refugia or support areas *	species, habitat, system	population, site, ecosystem
	Connectivity between different habitats *	species, system	ecosystem
**Process Attributes**	Connectivity to refugia areas	species, system	ecosystem
	Energy flows *	habitat, system	site, ecosystem
	Natural release from competition or predation	species	population
	Sedimentation	habitat, system	site, ecosystem
	Soil and air carbon balance	habitat	site
	Hyporheic flows	habitat, system	site, ecosystem
	Flow regime	species, system	site, ecosystem
	Groundwater contributions	habitat, system	site, ecosystem
	Structural legacies *	species, habitat	site
	Water infiltration	habitat	site
	Feedbacks between physical and biological processes	system	site, population, ecosystem
	Recovery (time) after disturbance	species, habitat	population, site
	Natural disturbance history *	species, habitat, system	site, ecosystem
	Random environmental variability	species, habitat, system	site, ecosystem
	Disturbance duration and intensity	species, habitat, system	site
	Degree of exposure to human pressures	habitat, system	site, ecosystem

Attributes classified according to methods described above.

* Attributes with 10 or more sources.

We illustrate how resilience metrics might be used in conservation or management of species or ecosystems with three applied examples. In each example, we focus on how practitioners might select a sub-set of resilience attributes for characterizing or monitoring resilience of species or ecosystems using the DST. The examples we selected—restoration of the Kissimmee River system in Florida, recovery of an endangered salmon population, and coral species restoration—demonstrate how a sub-set of resilience attributes and metrics differ depending on biological and management contexts and the scale at which attributes are measured.

#### Restoration at the ecosystem scale–the Kissimmee River example

The Kissimmee River once meandered for more than 100 miles through central Florida; connecting diverse habitats and supporting a thriving wetland ecosystem [185]. Restoration of the Kissimmee River System in Florida began two decades ago, and aims to reverse channelization and draining of wetlands to restore floodplain connectivity and restore ecosystem processes important to both the Kissimmee River and the Everglades ecosystem to which it drains. Based on the restoration focus (system) and scale (ecosystem) of the restoration effort, we derived 23 resilience attributes from the DST (Table 2) that are appropriate as restoration or monitoring variables. These attributes represent the community, ecosystem, and process categories (Table 3). Key resilience attributes within the community category are assemblage, diversity, redundancy, and connectivity. Not surprisingly, resilience attributes related to connectivity appear in all three major categories, as connectivity is a cornerstone of efforts to restore the Kissimmee River and Everglades ecosystem. In this case, each connectivity attribute increases resilience by allowing organisms and materials to move freely as suitable habitats shift in location. Within the ecosystem category, habitat area, condition, and variability are attributes that can support diversity or redundancy. Restoration efforts have largely focused on increasing natural habitat area and condition, including water quality and flow which are key metrics used to evaluate restoration success [185]. The remaining attributes in the process category tend to be features that also influence habitat condition and therefore support the community attributes. For example, energy flows is a broad and somewhat non-descript metric, however in this ecological context managers or restoration practitioners could consider (and already are) measuring how much water, sediment, and/or nutrients move between refuge areas.

**Table 3:** Kissimmee River restoration example.

	Resilience Attributes	Restoration Focus (species, habitat, system)	Scale of Application (population, site, ecosystem)
**Community Attributes**	Community structure	habitat, system	site, ecosystem
	Species assemblage *	habitat, system	site, ecosystem
	Species (alpha) diversity *	habitat, system	site, ecosystem
	Functional diversity *	habitat, system	site, ecosystem
	Response diversity	habitat, system	site, ecosystem
	Functional redundancy *	habitat, system	site, ecosystem
	Connectivity among communities	habitat, system	ecosystem
**Ecosystem Attributes**	Habitat area	species, habitat, system	population, site, ecosystem
	Habitat structure	species, habitat, system	site, ecosystem
	Habitat condition *	species, habitat, system	population, site, ecosystem
	Temporal variability in habitats *	system	ecosystem
	Spatial variability in habitats *	habitat, system	ecosystem
	Refugia or support areas *	species, habitat, system	population, site, ecosystem
	Connectivity between different habitats *	species, system	ecosystem
**Process Attributes**	Connectivity to refugia areas	species, system	ecosystem
	Energy flows *	habitat, system	site, ecosystem
	Sedimentation	habitat, system	site, ecosystem
	Hyporheic flows	habitat, system	site, ecosystem
	Groundwater contributions	habitat, system	site, ecosystem
	Feedbacks between physical and biological processes	system	site, population, ecosystem
	Natural disturbance history *	species, habitat, system	site, ecosystem
	Random environmental variability	species, habitat, system	site, ecosystem
	Degree of exposure to human pressures	habitat, system	site, ecosystem

Sub-set of resilience attributes for restoration focused on the system wide context at the ecosystem scale: Kissimmee River System.

* Attributes with 10 or more sources.

#### Restoration at the population scale–the pacific salmon Example

Recovery of salmon populations listed under the Endangered Species Act focuses on achieving several important targets, including adequate population size, population growth rate, spatial distribution, and diversity [186]. Each of these targets are listed in the resilience attributes’ population category and they are monitored and population performance is assessed using these criteria every 5 years. That is, these resilience attributes were selected to characterize recovery of salmon populations in part because they indicate both recovery of number of fish and recovery of population attributes that buffer populations against environmental change. This comports well with our DST, which suggests that relevant resilience attributes include genetic diversity and connectivity as well as growth, size, abundance, and life history flexibility in individuals and populations (Table 4). In addition, delisting criteria consider whether habitat factors contributing to listing have been abated. Consequently, various habitat-related resilience attributes are also appropriate for consideration in restoration planning or monitoring recovery. Habitat characteristics such as area, condition, and presence of refugia play an important role in the restoration of endangered populations and are often key components for the recovery of any species listed under the ESA. In addition to the metrics that align with current actions regarding salmon restoration, the DST provides several novel metrics that could be used to increase the resilience of endangered salmon populations to climate change, or to monitor changes in resilience among salmon populations.

**Table 4:** Pacific salmon restoration example.

	Resilience Attributes	Restoration Focus (species, habitat, system)	Scale of Application (population, site, ecosystem)
**Individual Attributes**	Individual growth rate	species	population
	Individual size	species	population
	Life span	species	population
	Individual characteristics that favor flexibility or adaptability *	species, habitat	population, site
	Reproductive strategy	species	population, site
	(Biological) Adaptation to disturbance *	species, habitat	population, site, ecosystem
	Presence of propagules	species, habitat	population, site
	Dispersal potential *	species, habitat	population, site
	Efficient water capture and use	species, habitat	population, site
**Population Attributes**	Genetic diversity *	species, habitat	population
	Population size *	species	population
	Population density *	species, habitat	population
	Population growth rate	species	population
	Population age structure	species	population
	Connectivity between populations*	species	population, ecosystem
**Ecosystem Attributes**	Habitat area	species, habitat, system	population, site, ecosystem
	Habitat condition *	species, habitat, system	population, site, ecosystem
	Refugia or support areas *	species, habitat, system	population, site, ecosystem
**Process Attributes**	Natural release from competition or predation	species	population
	Recovery (time) after disturbance	species, habitat	population, site

Sub-set of resilience attributes for species focused restoration at the population scale of application: Endangered Salmon Population.

* Attributes with 10 or more sources.

#### Restoration at the site scale–the coral reef example

One common management strategy for coral reef protection and restoration is the creation of Marine Protected Areas (MPA’s) [187]. For example, the Coral Triangle Initiative, a multi-lateral effort to address threats to reefs, fisheries, and food security in the South Pacific, is working towards establishing regional connectivity between MPA’s [188]. Restoration activities that focus on specific sites or habitats are more common for sessile species where the focus is either on restoring habitat for a species or ‘seeding’ a species to initiate recovery at a site and many of the following attributes resulting from the DST depend on having available habitat. Individual, ecosystem, and process categories are represented in the list of suitable resilience attributes for this type of restoration (Table 5). Individual attributes speak to a species’ ability to persist in an area. Ecosystem attributes are focused on habitat characteristics that may affect a species such as its condition, structure, or whether there are support areas present. Key process attributes that may affect habitat or species include structural legacies, disturbance, or degree of exposure to human pressures. Evidence suggests that conservation of sessile organisms such as coral reefs is most effective when an Ecosystem-based Management approach is taken. To address the many threats to coral reefs the creation of an MPA is coupled with land-based management to help reduce pollution sources [189].

**Table 5:** Coral reef restoration example.

	Resilience Attributes	Restoration Focus (species, habitat, system)	Scale of Application (population, site, ecosystem)
**Individual Attributes**	Individual characteristics that favor flexibility or adaptability *	species, habitat	population, site
	Reproductive strategy	species	population, site
	(Biological) Adaptation to disturbance *	species, habitat	population, site, ecosystem
	Presence of propagules	species, habitat	population, site
	Dispersal potential *	species, habitat	population, site
	Efficient water capture and use	species, habitat	population, site
**Ecosystem Attributes**	Habitat area	species, habitat, system	population, site, ecosystem
	Habitat structure	species, habitat, system	site, ecosystem
	Habitat condition *	species, habitat, system	population, site, ecosystem
	Refugia or support areas *	species, habitat, system	population, site, ecosystem
**Process Attributes**	Flow regime	species, system	site, ecosystem
	Structural legacies *	species, habitat	site
	Recovery (time) after disturbance	species, habitat	population, site
	Natural disturbance history *	species, habitat, system	site, ecosystem
	Random environmental variability	species, habitat, system	site, ecosystem
	Disturbance duration and intensity	species, habitat, system	site

Sub-set of resilience attributes for species focused restoration at the site scale of application: Coral Species Restoration.

* Attributes with 10 or more sources.

### Resilient restoration

Explicit consideration of climate change in restoration design is an increasingly common request among federal and state governmental agencies [15, 16, 190, 191], and many restoration projects are now required to evaluate the ability of a restored system or site to withstand impacts from climate change. Evidence suggests that when resilience is made an explicit planning objective, it offers a way to improve restoration projects as a whole [51, 102].

The purpose of our analysis is to assist restoration practitioners in identifying appropriate resilience attributes to measure and monitor within particular systems. The focus of the management or restoration action (species, habitat, or system) is the first basis for categorizing the resilience attributes, because the overarching goal or motivation of restoration will dictate objective setting and monitoring design. The scale at which the attributes should be measured is the second basis for selecting attributes. Together these two criteria can help distill a subset of potential resilience attributes that are suitable for a specific restoration action or monitoring efforts. The attributes and their associated metrics should be part of an adaptive management framework to be evaluated for their usefulness in conferring resilience to climate change.

## Conclusion

From our examination of recent ecological literature, we have extracted three key points that may be helpful in integrating resilience metrics into restoration plans. First, if made an explicit planning objective, as opposed to a component of existing objectives, resilience may be a way to improve restoration projects as a whole [51, 102]. By planning and monitoring for resilience, we are forced to identify sources of adaptive capacity within restored and natural ecosystems and to define actions that foster resilience. Second, considering the restoration focus and scale of a plan or project is essential in choosing appropriate resilience metrics to inform restoration efforts. In the face of climate change, restoration approaches that promote natural sources of resilience are more likely to be successful than those that focus on creating optimal steady states. Third, certain ecological attributes, such as diversity and connectivity, are more commonly considered to confer resilience because they apply to a wide variety of species and ecosystems. Even so, we identified numerous additional metrics that could potentially be useful for resilience planning.

The need to understand the dynamic nature of ecological systems, especially in the context of climate change, is crucial for successful restoration work. Improving our understanding of how certain ecological attributes confer resilience will help practitioners develop best practices for successful restoration in a changing climate. Past trends in climate and streamflow, for example, make it clear that stationarity of the physical environment is no longer a valid assumption in restoration planning. Moreover, we should not assume continuous directional change in ecosystems, as climate cycles and other sources of natural variability drive annual or decadal variation in habitats and species. Hence, assumptions made about response and recovery trajectories can greatly influence restoration planning decisions. By monitoring the response and recovery of a variety of species and ecosystems, we can better understand which attributes most contribute to ecological resilience to climate change.

## Supporting information

S1 Fig PRISMA 2009 flow diagram. (DOC)

S1 Table Interactive decision support table (DST). (XLSX)

S2 Table PRISMA 2009 checklist. (DOC)
