# Supplementary material for: A systematic review of ecological attributes that confer resilience to climate change in environmental restoration
Source: PLoS One. 2017 Mar 16;12(3):e0173812. doi: 10.1371/journal.pone.0173812 (PMC5354378; doi:10.1371/journal.pone.0173812)
Supplement: S1 Fig — (DOC) [file pone.0173812.s001.doc]

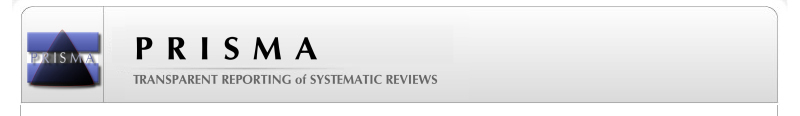
**PRISMA 2009 Flow Diagram**

**Screening**

**Included**

**Eligibility**

**Identification**

Records identified through database searching
(n =915 )

Additional records identified through other sources
(n =59 )

Records after duplicates removed
(n = 974 )

Records screened
(n = 291 )

Records excluded
(n = 683 )

Full-text articles assessed for eligibility
(n = 170 )

Full-text articles excluded, with reasons
(n = 0 )

Studies included in qualitative synthesis
(n = 170)

Studies included in quantitative synthesis (meta-analysis)
(n = 170 )
